# Efficacy and tolerability of bimatoprost versus travoprost in patients previously on latanoprost: a 3-month, randomised, masked-evaluator, multicentre study

**DOI:** 10.1136/bjo.2009.158071

**Published:** 2009-09-01

**Authors:** J A Kammer, B Katzman, S L Ackerman, D A Hollander

**Affiliations:** 1 Vanderbilt Eye Institute, Nashville, Tennessee, USA; 2 West Coast Eye Care Associates, San Diego, California, USA; 3 Philadelphia Eye Associates, Philadelphia, Pennsylvania, USA; 4 Allergan, Irvine, California, USA

## Abstract

**Aim:**

To evaluate the efficacy and safety of replacing latanoprost with another prostaglandin analogue (PGA) in patients with glaucoma or ocular hypertension requiring additional intraocular pressure (IOP) lowering while on latanoprost.

**Methods:**

Prospective, randomised, investigator-masked, multicentre clinical trial. Patients on latanoprost 0.005% monotherapy requiring additional IOP lowering discontinued latanoprost and were randomised to bimatoprost 0.03% (n = 131) or travoprost 0.004% (n = 135). IOP was measured at latanoprost-treated baseline and after 1 month and 3 months of replacement therapy.

**Results:**

Baseline mean diurnal IOP on latanoprost was similar between groups. The mean diurnal IOP was significantly lower with bimatoprost than with travoprost at 1 month (p = 0.009) and 3 months (p = 0.024). Overall, 22.0% of bimatoprost patients versus 12.1% of travoprost patients achieved a ⩾15% reduction in diurnal IOP from latanoprost-treated baseline at both months 1 and 3 (p = 0.033). At month 3, the additional mean diurnal IOP reduction from latanoprost-treated baseline was 2.1 (95% CI 1.7 to 2.5) mm Hg (11.0%) with bimatoprost and 1.4 (95% CI 0.9 to 1.8) mm Hg (7.4%) with travoprost (p = 0.024). At 3 months, 11.5% of bimatoprost and 16.5% of travoprost patients demonstrated a ⩾1-grade increase in physician-graded conjunctival hyperaemia (p = 0.288). Hyperaemia was reported as a treatment-related adverse event in 3.1% of bimatoprost and 1.5% of travoprost patients (p = 0.445).

**Conclusion:**

Patients on latanoprost requiring lower IOP achieved a greater additional short-term diurnal IOP reduction when latanoprost was replaced by bimatoprost compared with travoprost. Low rates of hyperaemia were observed in patients treated with bimatoprost or travoprost after switching from latanoprost.

A primary goal of medical therapy in glaucoma is to reduce intraocular pressure (IOP). The once-daily prostaglandin analogues (PGAs; bimatoprost, latanoprost and travoprost) typically provide significant reductions in IOP^1^ ^2^ ^3^ ^4^ and have become the most commonly used first-line agents in glaucoma and ocular hypertension (OHT).^5^ In achieving and maintaining a target IOP, monotherapy is typically preferred over the use of multiple IOP-lowering medications to ensure better compliance and minimise the risk of topical and systemic adverse effects.

Patients on a particular PGA who require additional IOP lowering may achieve further IOP reduction when switched to a different topical medication within the PGA class. Data from parallel-group, randomised clinical trials suggest that bimatoprost and possibly travoprost may reduce IOP more effectively than latanoprost,^3^ ^6^ ^7^ ^8^ ^9^ with a difference in efficacy between latanoprost and travoprost apparent in late-afternoon IOP measurements.^3^ ^9^ In patients who are poorly responsive or non-responsive to latanoprost therapy, bimatoprost has been demonstrated to provide significant IOP lowering.^10^

Studies have demonstrated that even patients who respond to latanoprost therapy may achieve additional IOP lowering upon changing therapy to another PGA.^11^ ^12^ ^13^ ^14^ Increased efficacy was observed on a population level when patients in a managed care plan were systematically switched from latanoprost to bimatoprost therapy following changes in the HMO’s pharmacy formulary.^11^ In contrast, the mean IOP of 84 patients systematically switched from latanoprost to travoprost in a prospective study was unchanged up to 3 months after the switch to travoprost.^15^ Open-label studies have shown significant additional mean IOP lowering of 3.5 mm Hg or 3.2 mm Hg when latanoprost was replaced by bimatoprost^12^ or travoprost,^13^ ^14^ respectively.

Regardless of the IOP-lowering response to latanoprost, the literature suggests that patients who fail to achieve sufficiently low IOP on latanoprost may obtain additional IOP lowering when switched to another PGA. The purpose of the present study was to evaluate the efficacy and tolerability of replacing latanoprost monotherapy with either bimatoprost or travoprost monotherapy when IOP was not sufficiently reduced by latanoprost alone.

## Methods

This randomised, multicentre, investigator-masked, parallel-group clinical study compared bimatoprost 0.03% with travoprost 0.004% as replacement therapy for patients on latanoprost who required additional IOP lowering. The study was approved by the Institutional Review Board at each of the 17 sites and was carried out in accordance with HIPAA regulations. All patients who participated in the study provided written informed consent. The study was registered on the clinicaltrials.gov website with the identifier NCT00440011.

The study enrolled adult patients diagnosed as having glaucoma or OHT in each eye who in the judgement of the investigator had inadequate IOP control after at least 30 days on latanoprost monotherapy. Patients were also required to have best-corrected visual acuity equivalent to a Snellen score of 20/100 or better in each eye. Primary exclusion criteria included previous inadequate IOP response to bimatoprost or travoprost, known hypersensitivity or contraindication to any component of the study medications, active intraocular inflammation or macular oedema that might be exacerbated by study treatment, and intraocular or corneal refractive surgery within 3 months prior to study entry.

After a screening visit, eligible patients were continued on latanoprost monotherapy for an additional 2 weeks until the baseline visit (day 0). At baseline, patients discontinued latanoprost and were randomised 1:1 to monotherapy with either bimatoprost 0.03% (Lumigan, Allergan, Irvine, California) or travoprost 0.004% (Travatan, Alcon Laboratories, Fort Worth, Texas). The randomisation code was computer-generated, and treatment assignments were unavailable to the investigators during the study. To maintain investigator masking, bottles of the study drugs were provided to patients in identically appearing masked cartons labelled with the patient randomisation number, and patients were instructed not to disclose the study medication to the investigator or office staff. Patients were instructed to instill one drop of the study medication in each eye once daily in the evening and were scheduled for follow-up visits at months 1 and 3.

The primary efficacy outcome measures were mean IOP at each time point and mean diurnal IOP. IOP was measured using a calibrated Goldmann applanation tonometer at 09:00 and 16:00 (±1 h) at the baseline, month 1 and month 3 visits. For each eye, the average of two consecutive measurements was recorded.

Safety outcome measures included ocular signs on slit-lamp biomicroscopy, adverse events and visual acuity. Biomicroscopic findings were scored on a four-grade scale of none to trace (0 to 0.5), mild (1), moderate (2) and severe (3). An adverse event was defined as any new condition, worsening of a pre-existing condition or recurrence of a condition that had resolved after the baseline visit. All adverse events observed by the investigator or reported by patients at the month 1 and month 3 study visits were recorded, and their severity and potential relationship to study treatment were documented.

The preplanned analyses of IOP were based on the worse eye (the eye with the higher IOP at 09:00 on baseline) for the intent-to-treat patient population (all randomised patients) with no imputation for missing values and used analysis of variance (ANOVA) to test baseline differences between treatment groups and analysis of covariance (ANCOVA) with baseline IOP as the covariate to test differences between treatment groups at follow-up. The parameters evaluated were mean IOP at each hour, mean diurnal IOP at each visit, and mean change from baseline diurnal IOP and IOP at each hour. Diurnal IOP was calculated as the average of the 09:00 and 16:00 measurements in the study eye at the particular visit. The a priori statistical plan for the study included a subgroup analysis for patients whose baseline IOP (at 09:00) on latanoprost was ⩾20 mm Hg.

Categorical variables were analysed using the χ^2^ test or Fisher exact test. Continuous variables were analysed using ANOVA or ANCOVA. The percentage of patients with increased biomicroscopic scores was compared between groups using the Cochrane–Mantel–Haenszel row mean-score test.

All statistical tests were two-tailed with an alpha level of 0.05. Statistical analyses were performed using SAS version 8.2 (SAS Institute, Cary, North Carolina). The planned sample size was 125 patients in each group, which provided 80% power to detect a 1 mm Hg difference between groups, assuming a common SD of 2.8 mm Hg.

## Results

### Patient baseline characteristics and disposition

Two hundred and sixty-six patients with inadequate IOP control on latanoprost monotherapy were enrolled in the study and randomised to either bimatoprost or travoprost monotherapy. Patient characteristics at baseline are listed in table 1. A larger percentage of patients in the travoprost group were male. There were no other statistically significant differences in demographics between treatment groups. Most patients were diagnosed as having bilateral glaucoma associated with elevated IOP.

**Table 1:** Patient characteristics at baseline

	Bimatoprost (n = 131)	Travoprost (n = 135)	p Value
Mean (SD) age in years, range	63.4 (12.3), 27 to 91	62.7 (12.4), 19 to 88	0.609
Sex			**0.034**
Female	81 (61.8%)	66 (48.9%)	
Male	50 (38.2%)	69 (51.1%)	
Race			0.572*
Black or African–American	36 (27.5%)	33 (24.4%)	
White	75 (57.3%)	80 (59.3%)	
Hispanic or Latino	16 (12.2%)	15 (11.1%)	
Asian	2 (1.5%)	4 (3.0%)	
American Indian or Alaska Native	0 (0.0%)	2 (1.5%)	
Native Hawaiian or Pacific Islander	2 (1.5%)	0 (0.0%)	
Multiracial	0 (0.0%)	1 (0.7%)	
Iris colour			0.950†
Brown	82 (62.6%)	84 (62.2%)	
Blue	30 (22.9%)	31 (23.0%)	
Hazel	12 (9.2%)	16 (11.9%)	
Green	7 (5.3%)	4 (3.0%)	
Diagnosis			0.644
Ocular hypertension (OHT)	28 (21.4%)	29 (21.5%)	
Primary open-angle glaucoma (POAG) or	101 (77.1%)	99 (73.3%)	
pigmentary glaucoma			
Chronic angle-closure glaucoma (CACG)	0 (0.0%)	1 (0.7%)	
Mixed‡	1 (0.8%)	3 (2.2%)	
Other§	1 (0.8%)	3 (2.2%)	

Value shown in bold: p<0.05.

*p Value for black or African–American versus all other race categories.

†p Value for dark (brown) versus light (blue, hazel or green).

‡One eye diagnosed as having OHT and the other with open-angle glaucoma.

§Pseudoexfoliative glaucoma or normal-tension glaucoma.

Study completion rates were high in each group, and 259 patients (97.4%) completed the study (table 2).

**Table 2:** Patient disposition

	Bimatoprost	Travoprost
Patients randomised	131 (100.0%)	135 (100.0%)
Completed study	127 (96.9%)	132 (97.8%)
Discontinued	4 (3.1%)	3 (2.2%)
Reason for discontinuation		
Adverse event*	2 (1.5%)	0 (0.0%)
Protocol violation	1 (0.8%)	1 (0.7%)
Lost to follow-up	0 (0.0%)	2 (1.5%)
Personal reason	1 (0.8%)	0 (0.0%)

*Adverse event could be related or unrelated to treatment.

### IOP-lowering efficacy

Intraocular pressure data were available for 98.7% (1575/1596) of planned measurements. At the latanoprost-treated baseline, there were no significant differences between treatment groups in mean IOP at individual time points (09:00 and 16:00) or in mean (SD) diurnal IOP (bimatoprost: 19.1 (SD 2.8) mm Hg; travoprost: 18.9 (2.7) mm Hg; p = 0.473). After replacement of latanoprost therapy with bimatoprost or travoprost therapy, the mean IOP was significantly lower with bimatoprost than with travoprost at the 09:00 time point at month 1 and the 16:00 time point at month 3 (table 3).

**Table 3:** Mean intraocular pressure (IOP) at each hour and visit

	Baseline		Month 1		Month 3	
	09:00	16:00	09:00	16:00	09:00	16:00
Bimatoprost						
Mean (SD) IOP, mm Hg	19.8 (2.9)	18.5 (3.1)	17.6 (2.8)	16.8 (2.8)	17.6 (3.3)	16.5 (3.2)
n	131	131	129	128	128	128
Travoprost						
Mean (SD) IOP, mm Hg	19.5 (3.1)	18.2 (2.9)	18.3 (3.1)	17.0 (2.7)	18.1 (3.0)	17.0 (2.7)
n	135	135	133	133	132	132
p Value	0.530	0.492	**0.004**	0.162	0.058	**0.047**

Values shown in bold: p<0.05.

The mean reduction from baseline IOP on latanoprost was up to 2.2 mm Hg (11.1%) with bimatoprost and up to 1.5 mm Hg (7.7%) with travoprost at the 09:00 measurements and up to 2.0 mm Hg (10.8%) with bimatoprost and up to 1.2 mm Hg (6.6%) with travoprost at the 16:00 measurements (table 4).

**Table 4:** Mean decrease from baseline intraocular pressure (IOP)

	Month 1			Month 3		
	09:00	16:00	Diurnal	09:00	16:00	Diurnal
Bimatoprost						
Mean (SD) IOP reduction, mm Hg	2.2 (2.5)	1.7 (2.6)	1.9 (2.2)	2.2 (2.8)	2.0 (2.7)	2.1 (2.4)
Percentage reduction	11.1%	9.2%	9.9%	11.1%	10.8%	11.0%
n	129	128	129	128	128	128
Travoprost						
Mean (SD) IOP reduction, mm Hg	1.2 (2.8)	1.2 (2.7)	1.2 (2.3)	1.5 (3.1)	1.2 (2.9)	1.4 (2.5)
Percentage reduction	6.2%	6.6%	6.3%	7.7%	6.6%	7.4%
n	133	133	133	132	132	132
p Value	**0.004**	0.162	**0.009**	0.058	**0.047**	**0.024**

Values shown in bold: p<0.05.

**Figure 1:**
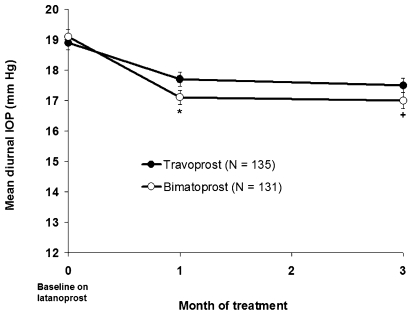
Mean diurnal intraocular pressure (IOP) in each treatment group at latanoprost-treated baseline and after 1 and 3 months of bimatoprost or travoprost replacement therapy. Error bars represent the standard error of the mean. *p = 0.009 versus travoprost; ^+^p = 0.024 versus travoprost.

The mean diurnal IOP was significantly lower with bimatoprost than with travoprost at both months 1 and 3 (fig 1). Replacing latanoprost with travoprost led to a mean reduction from baseline diurnal IOP of 1.2 (95% CI 0.8 to 1.6) mm Hg at month 1 and 1.4 (95% CI 0.9 to 1.8) mm Hg at month 3, while replacing latanoprost with bimatoprost led to significantly larger mean decreases from baseline diurnal IOP of 1.9 (95% CI 1.6 to 2.3) mm Hg at month 1 and 2.1 (95% CI 1.7 to 2.5) mm Hg at month 3 (table 4).

**Figure 2:**
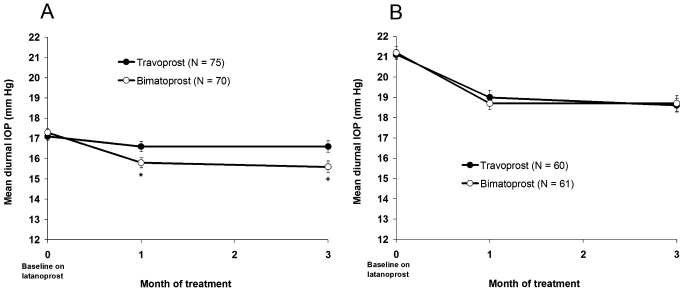
Mean diurnal intraocular pressure (IOP) at latanoprost-treated baseline and after 1 and 3 months of bimatoprost or travoprost replacement therapy for patients with baseline IOP<20 mm Hg (A) and patients with baseline IOP⩾20 mm Hg (B). Error bars represent standard error of the mean. *p = 0.007 versus travoprost; ^+^p<0.001 versus travoprost.

In the subgroup of patients whose baseline IOP on latanoprost was <20 mm Hg, the mean diurnal IOP was significantly lower with bimatoprost than with travoprost at both 1 and 3 months (fig 2A). At month 3, the mean reduction from latanoprost-treated baseline diurnal IOP was 1.8 (95% CI 1.3 to 2.2) mm Hg (10.4%) with bimatoprost versus 0.5 mm (95% CI 0.0 to 1.0) Hg (2.9%) with travoprost (p<0.001). In contrast, there was no significant difference in efficacy between bimatoprost and travoprost in the subgroup of patients whose baseline IOP on latanoprost was ⩾20 mm Hg (fig 2B). At month 3, the mean reduction from latanoprost-treated baseline diurnal IOP in these patients was 2.5 (95% CI 1.7 to 3.2) mm Hg (11.8%) for bimatoprost and 2.5 (95% CI 1.8 to 3.2) mm Hg (11.8%) for travoprost.

Overall, an additional reduction in diurnal IOP from latanoprost-treated baseline of at least 15% was achieved at both month 1 and month 3 by 22.0% of patients in the bimatoprost group compared with 12.1% of patients in the travoprost group (p = 0.033). In subgroup analyses, at least a 15% reduction in diurnal IOP from latanoprost-treated baseline was achieved at both month 1 and month 3 by 20.3% of bimatoprost patients versus 8.2% of travoprost patients who had baseline diurnal IOPs<20 mm Hg (p = 0.053) and by 24.1% of bimatoprost patients versus 16.9% of travoprost patients who had baseline diurnal IOPs⩾20 mm Hg (p = 0.368).

### Safety and tolerability

Both bimatoprost and travoprost were well tolerated and associated with a low incidence of adverse events. Only two patients, both in the bimatoprost group, discontinued from the study early because of adverse events (periorbital itching and dryness; asthma exacerbation unrelated to treatment). Treatment-related adverse events (table 5) were reported for 11 patients (8.4%) in the bimatoprost group and eight patients (6.0%) in the travoprost group (p = 0.485). None were categorised as serious. Ocular or conjunctival hyperaemia was reported as a treatment-related adverse event for 3.1% of bimatoprost patients and 1.5% of travoprost patients (p = 0.445).

**Table 5:** Treatment-related adverse events

Adverse event	No (%) of patients	
	Bimatoprost (n = 131)	Travoprost (n = 133)
Eye pruritus	3 (2.3%)	1 (0.8%)
Conjunctival or ocular hyperaemia	4 (3.1%)	2 (1.5%)
Punctate keratitis	1 (0.8%)	2 (1.5%)
Eye irritation	2 (1.5%)	0 (0.0%)
Growth of eyelashes	2 (1.5%)	0 (0.0%)
Abnormal sensation in eye	1 (0.8%)	0 (0.0%)
Drug hypersensitivity	0 (0.0%)	1 (0.8%)
Dry skin	1 (0.8%)	0 (0.0%)
Foreign-body sensation	0 (0.0%)	1 (0.8%)
Hyperaemia (non-ocular)	0 (0.0%)	1 (0.8%)
Pruritus at instillation site	0 (0.0%)	1 (0.8%)
Skin pigmentation	1 (0.8%)	0 (0.0%)
Visual acuity reduced	0 (0.0%)	1 (0.8%)
Overall*	11 (8.4%)	8 (6.0%)

*Patients reporting any treatment-related adverse event.

**Figure 3:**
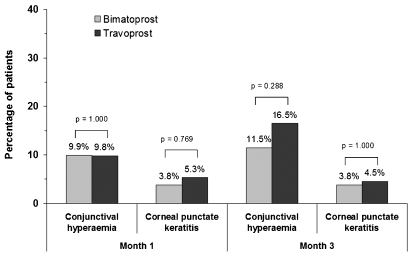
Percentage of patients with at least a one-grade increase from baseline in biomicroscopic conjunctival hyperaemia and corneal punctate keratitis scores. Most patients in each group had none-to-trace conjunctival hyperaemia and punctate keratitis on latanoprost baseline (bimatoprost: 93%, 96%; travoprost: 88%, 95%, respectively).

On biomicroscopy, conjunctival hyperaemia and punctate keratitis were the only findings with ⩾1-grade increases in severity reported in at least 4% of patients in either treatment group (fig 3). At 3 months, the percentages of patients with a one-grade, two-grade or three-grade increase in the severity of conjunctival hyperaemia from baseline, respectively, were 8.4%, 2.3% and 0.8% in the bimatoprost group and 13.5%, 3.0% and 0.0% in the travoprost group. There were no significant differences between treatment groups, and none of the patients in the study discontinued due to conjunctival hyperaemia or punctate keratitis.

There was no significant between-group difference in the change from baseline visual acuity.

## Discussion

The primary goal of glaucoma treatment is to reduce IOP to the target pressure using a minimal number of medications.^16^ This prospective, randomised study confirms previous reports^10^ ^12^ ^13^ ^14^ demonstrating that additional IOP lowering may be achieved by switching patients who are inadequately controlled on latanoprost to another PGA and reinforces the concept that changing therapy within the PGA class should be considered prior to adding a second medication if further IOP lowering is required.^17^

Despite the fact that the mean diurnal IOP on latanoprost was approximately 19 mm Hg at baseline in both treatment groups, which is within the statistically normal range of IOPs, patients still achieved additional IOP lowering after switching to either bimatoprost or travoprost. The mean diurnal IOP was significantly lower with bimatoprost than with travoprost at both 1 and 3 months. It has been suggested that an adjunctive medication should provide at least 15% additional IOP lowering.^17^ In this study, 22% of patients consistently achieved at least 15% additional IOP lowering after replacing latanoprost with bimatoprost, that is, they had as much additional IOP lowering as if they had successfully added a second medication to latanoprost, yet they were able to maintain monotherapy.

Conjunctival hyperaemia, the most common side effect of the PGAs, has typically been reported as an unsolicited adverse event in 40% to 50% of patients treated with bimatoprost or travoprost after washout of prior medications.^1^ ^3^ ^6^ In the present study, a low percentage of patients had increased conjunctival hyperaemia after switching from latanoprost (10% in each treatment group at month 1). This study more closely replicates clinical practice in that patients were switched directly from latanoprost without undergoing a washout. In fact, there were only four adverse event reports of treatment-related hyperaemia in the bimatoprost group and two in the travoprost group. More importantly, no patients discontinued using either medication due to conjunctival hyperaemia. The tolerability findings are consistent with prior reports in which patients were systematically switched from latanoprost to another PGA,^11^ illustrating that prior PGA exposure and a direct switch among PGAs reduce the incidence and severity of hyperaemia associated with bimatoprost or travoprost.

The decision of whether to switch medications or add another medication can be difficult when patients’ IOPs are relatively controlled (eg, less than 20 mm Hg) on monotherapy. The results of the subgroup analysis indicate that replacing latanoprost with bimatoprost is a viable option, even for these patients. For patients whose baseline IOP on latanoprost was less than 20 mm Hg, bimatoprost provided 1.8 mm Hg of additional diurnal IOP lowering compared with the 0.5 mm Hg provided by travoprost (p<0.001). In contrast, bimatoprost and travoprost were similarly effective in patients whose baseline IOP on latanoprost was 20 mm Hg or higher, each reducing diurnal IOP by 2.5 mm Hg after 3 months of treatment.

Meta-analyses of data from parallel-group^8^ and 24 h crossover^18^ clinical studies have suggested that bimatoprost and travoprost are the most effective medications available for IOP lowering. In head-to-head studies comparing bimatoprost and travoprost in treatment-naïve patients or patients washed out of previous medications, bimatoprost has reduced IOP at least as well or significantly better than travoprost.^19^ ^20^ ^21^ ^22^ These results are consistent with the present study, which demonstrated a substantial decrease in mean IOP when latanoprost was replaced with either bimatoprost or travoprost. In addition, the mean IOP was significantly lower with bimatoprost than with travoprost at half of the time points.

The difference in efficacy between bimatoprost and latanoprost in the present study was similar in magnitude to that in the comparison study reported by Noecker and associates,^6^ yet larger than the average difference of approximately 1 mm Hg that has been reported.^7^ ^8^ This discrepancy may be explained in part by differences in study populations and study design, as patients in previous studies were typically treatment-naïve or washed out of previous medications rather than switched directly between the PGAs. In the present study, all patients were treated with latanoprost prior to randomisation to masked treatment with bimatoprost or travoprost, potentially introducing an element of bias in the comparisons of latanoprost with the other PGAs.

Interpretation of the results of switch studies is sometimes limited by the possibility of an increase in compliance. A change in compliance is unlikely to account for the IOP reduction observed in the present study, however, as patients who were screened were required to be run-in on latanoprost for an additional 2 weeks prior to the baseline evaluations. The present study was limited because tests of within-group changes from baseline IOP were not included in the a priori statistical plan for the study. However, posthoc analyses using within-group t tests showed that the changes from baseline diurnal IOP were statistically significant in both the bimatoprost and travoprost groups (p<0.001). Of note, the reduction in IOP was statistically significant for bimatoprost in patients with baseline IOP<20 mm Hg or ⩾20 mm Hg (p<0.001). In contrast, in patients with baseline IOP<20 mm Hg who switched to travoprost treatment, the change from baseline IOP did not achieve statistical significance at 3 months (p = 0.187). As the inclusion criteria only required at least 30 days of latanoprost therapy without any further delineation or stratification based on length of prior use, we are unable to relate the duration of latanoprost treatment to outcomes in the present study. An additional limitation was that this was a short-term study, and diurnal IOP was determined based on only two time points over a period of time representing a typical office day. Future studies of longer duration or using additional time points will be needed to fully evaluate efficacy during long-term treatment and over the course of a 24 h period.

In summary, switching therapy within the PGA class may allow patients to reach lower pressures while maintaining monotherapy. The results of this study have demonstrated that when additional efficacy is needed, switching from latanoprost monotherapy to bimatoprost may provide greater additional IOP lowering than switching to travoprost. The rate of increased hyperaemia is low in patients switched directly from latanoprost to either bimatoprost or travoprost.
